# Genetic variations at the human *growth hormone receptor (GHR)* gene locus are associated with idiopathic short stature

**DOI:** 10.1111/jcmm.13210

**Published:** 2017-05-29

**Authors:** Christel Dias, Mara Giordano, Rosalie Frechette, Simonetta Bellone, Constantin Polychronakos, Laurent Legault, Cheri L Deal, Cynthia Gates Goodyer

**Affiliations:** ^1^ Department of Experimental Medicine McGill University Montreal QC Canada; ^2^ Laboratory of Human Genetics Department of Health Science University of Eastern Piedmont Novara Italy; ^3^ Genotyping Platform Genome‐Québec Montreal QC Canada; ^4^ Division of Pediatrics Department of Health Science University of Eastern Piedmont Novara Italy; ^5^ Departments of Experimental Medicine, Human Genetics and Pediatrics McGill University Montreal QC Canada; ^6^ Department of Pediatrics McGill University Montreal QC Canada; ^7^ CHU Ste‐Justine Research Centre and Department of Pediatrics Université de Montréal Montreal QC Canada; ^8^ Departments of Experimental Medicine and Pediatrics McGill University Montreal QC Canada

**Keywords:** growth hormone receptor, idiopathic short stature, short stature, GT microsatellite, SNPs, haplotype

## Abstract

GH plays an essential role in the growing child by binding to the growth hormone receptor (GHR) on target cells and regulating multiple growth promoting and metabolic effects. Mutations in the *GHR* gene coding regions result in GH insensitivity (dwarfism) due to a dysfunctional receptor protein. However, children with idiopathic short stature (ISS) show growth impairment without GH or GHR defects. We hypothesized that decreased expression of the *GHR* gene may be involved. To test this, we investigated whether common genetic variants (microsatellites, SNPs) in regulatory regions of the *GHR* gene region were associated with the ISS phenotype. Genotyping of a GT‐repeat microsatellite in the *GHR* 5′UTR in a Montreal ISS cohort (*n* = 37 ISS, *n* = 105 controls) revealed that the incidence of the long/short (L/S) genotype was 3.3× higher in ISS children than controls (*P* = 0.04, OR = 3.85). In an Italian replication cohort (*n* = 143 ISS, *n* = 282 controls), the medium/short (M/S) genotype was 1.9× more frequent in the male ISS than controls (*P* = 0.017, OR = 2.26). In both ISS cohorts, logistic regression analysis of 27 SNPs showed an association of ISS with rs4292454, while haplotype analysis revealed specific risk haplotypes in the 3′ haploblocks. In contrast, there were no differences in GT genotype frequencies in a cohort of short stature (SS) adults *versus* controls (CARTaGENE: *n* = 168 SS, *n* = 207 controls) and the risk haplotype in the SS cohort was located in the most 5′ haploblock. These data suggest that the variants identified are potentially genetic markers specifically associated with the ISS phenotype.

## Introduction

The growth hormone (GH)‐insulin‐like growth factor 1(IGF‐1) axis is recognized as a key regulator of normal musculoskeletal development in the child. If any member of this axis is defective, the result is a short stature phenotype 1. Loss‐of‐function mutations in the *growth hormone receptor* (*GHR*) gene are a prime example because the ability of GH to exert its pleiotropic effects is contingent on the availability of its receptor at the surface of target cells 2. Individuals with a dysfunctional GHR or loss of GHR do not respond normally to GH: they are not only extremely short, and they have decreased bone mineral density and increased adiposity, with a greater risk of osteoporosis, lipid disorders and cardiovascular disease 3, 4, 5.

However, in ~60–80% of childhood short stature cases (defined as a height *z*‐score below −2 S.D.), no aetiology can be found; the children have normal birth length and are GH sufficient and there is no evidence of systemic, endocrine, nutritional or chromosomal abnormalities 6, 7. These individuals are defined as having idiopathic short stature (ISS). This is a heterogeneous population. Approximately half will be subcategorized as having ‘constitutional growth delay’: their pubertal development and growth spurt are delayed but, when they do occur, due to increased production of sex steroids, average final height is achieved 1, 7. A smaller percentage will be diagnosed with familial short stature as their predicted final heights are within the expected range for parental target height. The remaining children, who never achieve a ‘catch‐up’ growth and who have limited responsiveness to GH therapy, represent ~1–2% of populations worldwide 8, 9, 10.

One previous explanation for ISS was *GHR* haploinsufficiency. However, when the *GHR* coding exons were examined for heterozygous deleterious mutations, only 2–5% of ISS individuals were found to have coding sequence changes and most of these were not functionally significant 11, 12, 13. On the other hand, serum levels of GH binding protein, the product of enzymatic cleavage of cell‐surface GHR, are often low and ~20% of ISS children have levels >2 S.D. below the mean, suggesting that tissue levels of GHR are chronically low. Additional evidence comes from studies of the African Baka pygmies. These individuals, who have a similar phenotype and endocrine profile as the ISS children, show a ~80% decrease of *GHR* mRNA in their lymphocytes 14. Together these data suggest low transcription of the *GHR* gene may be occurring.

Height is a complex polygenic trait with a high heritability estimate (h^2^~0.80–0.90) 15, 16. Since 2007, an increasing number of gene variants have been shown to be associated with height variation in the general population 17, 18, 19, 20, 21, 22. The most recent GWAS identified 697 common variants that explain ~16% of the adult height variation and implicate many genes and pathways important for skeletal growth 22. Surprisingly, the early reports, including the initial study from the GIANT consortium, did not find any association of common genetic variants in *GH/IGF‐1* axis genes with adult height variation 17, 21, 23. Only the later use of more dense gene‐centric arrays revealed significant associations with some axis members, including *GHR*: two SNPs, rs17574650 in intron 1 (MAF < 3%) and rs6180 in exon 10, were identified within the *GHR* locus 22, 24.

One explanation for the relative lack of detection of the *GHR* gene is that sampling of population height in previous studies has generally excluded the extreme tails of the normal distribution. In addition, most of the common variants identified through GWAS studies have a small effect size and, thus, cannot explain all of the height variation. To understand this ‘missing heritability’, other classes and possible combinations of genetic variations need to be explored, including microsatellites 25, 26. Microsatellite polymorphisms are a ubiquitous class of simple repetitive DNA sequences 27. For example, the (GT)_n_ repeat is frequently observed in the human genome; population studies show high mutability due to slippage, leading to complex polymorphic characteristics 28, 29. Interestingly, the length of the GT repeat in promoter regions has been shown to modulate flanking response elements in several genes 30, 31, 32, 33, 34, 35. We previously reported that the GT repeat in the *GHR* V9 promoter region is a microsatellite polymorphism, with 19–32 repeats in the general population 36.

In this study, we have tested the hypothesis that GHR, because of its important position within the GH/IGF‐1 axis, has a role in the occurrence of ISS. To do this, we have analysed both the V9 microsatellite polymorphism as well as multiple SNPs within the *GHR* locus in two ISS cohorts and a cohort of short stature (SS) adults, along with their respective controls, to determine whether there is a *GHR* regulatory haplotype associated with the ISS phenotype.

## Materials and methods

### Study populations

The Montreal ISS cohort was comprised of 37 ISS children recruited from the Montreal Children's Hospital (MCH) and l'Hôpital Ste‐Justine (HSJ) from 2009 to 2010 (Table 1). The major ISS inclusion criterion was a height score (SDS or *Z*‐score) ≤−2 S.D. and normal stimulated (clonidine, arginine) GH levels (cut‐off values of 5 [MCH] and 6.5 [HSJ] μg/L), with no evidence of organic disease, malnutrition, psychosocial issues, intrauterine growth retardation (IUGR) or hypothyroidism. We also obtained genomic DNA from 105 adults with normal final adult heights; these individuals were initially enrolled in the Type I Diabetes Susceptibility Study but are non‐diabetic. Exclusion criteria for the adults included IUGR, small for gestational age (SGA), a childhood comorbid disease or specific syndromes.

**Table 1:** Baseline characteristics of study participants

	Montreal cohort			Novara cohort		CARTaGENE cohort	
	ISS	Controls	Controls†	ISS	Controls	SS	Controls
Total number of individuals	37	105	57	143	282	168	207
Sex							
Males	23	36	16	76	159	92	108
Females	14	69	41	67	123	76	99
Age‡ (years)							
Median (range)	9.91 (2.5, 14.5)	43 (28, 60)	42.5 (28, 58)	10.9 (5.1, 17.8)§		59.34 (41.1, 69.5)	57.85 (40.3, 70.1)
Height *z*‐score¶ (SDS)							
Median (range)	−2.49 (−4.56, −1.84)	−0.02 (−1.83, 1.57)	0.01 (−0.55, 0.57)	−2.2 (−4.6, −1.5)	n/a	−2.675 (−3.83, −2.4)	0.01 (−0.49, 0.59)
Males	−2.41 (−3.81, −1.84)	−0.085 (−1.36, 1.57)	−0.01 (−0.55, 0.49)	−2.2 (−3.5, −1.5)	n/a	−2.615 (−3.83, −2.4)	−0.08 (−0.49, 0.47)
Females	−2.8 (−4.56, −2.09)	−0.02 (−1.83, 1.38)	0.02 (−0.54, 0.57)	−2.2 (−4.6, −1.8)	n/a	−2.49 (−3.8, −2.47)	0.08 (−0.48, 0.59)

n/a, not available; ISS, idiopathic short stature. ^†^Montreal adult control group restricted to heights matching the CARTaGENE adult controls (30th–70th percentile). ^‡^Age at diagnosis for the two ISS cohorts and age at enrolment for the CARTaGENE cohort. ^§^Median (range) age for Novara ISS and control groups (age‐matched controls) 34. ^¶^Height *z*‐scores (Standard Deviation Scores) were calculated using the World Health Organization Growth Charts for Canada (2014). Height *z*‐scores were established at the time of diagnosis for the two ISS paediatric cohorts and at enrolment for the CARTaGENE adult cohort.

Genomic DNA samples from the Novara cohort have been described elsewhere 37. Briefly, 143 ISS children, along with 282 normal stature healthy children matched for age and sex, were recruited by the Unit of Paediatrics of the Department of Health Science of Novara (Italy) (Table 1). Criteria for ISS diagnosis were similar to the Montreal ISS cohort, including a height SDS ≤−2 and GH sufficiency (cut‐off value of 8 μg/l).

CARTaGENE is the largest population biobank in Quebec, with ~20,000 recruits aged 40–69 years at the time that our study was initiated (www.cartagene.qc.ca) 38; the participants represent a random selection of individuals residing in the metropolitan areas of Quebec. Our CARTaGENE cohort consisted of 168 SS individuals with a final height corresponding to severe short stature (males: maximum height of 159 cm, −2.4 SDS; females: maximum height of 147 cm, −2.47 SDS) and 207 controls of average height (30^th^–70^th^ percentile [~±0.5 SDS]: males were 173−180 cm; females were 160–167 cm) (Table 1). There were no data available on whether the SS individuals had ever been diagnosed with ISS.

Recruitment of these patients was approved by local institutional review boards, including the Azienda Ospedaliera Universitaria Maggiore della Carita for the Italian cohort, the Research Ethics Committee of CHU Ste‐Justine for CARTaGENE participants and the Research Ethics Boards at both CHU Ste‐Justine and the McGill University Health Centre for the Montreal ISS cohort. In all cases, information on the recruits was anonymized prior to receiving the DNA samples for analysis. The majority (>95%) of the participants in the three cohorts were from European ancestry. Asian, African and South American individuals were excluded to minimize stratification of the genetic results.

Analyses were also carried out on a pool of the two ISS cohorts. In addition, we created a pool of Montreal and CARTaGENE adult controls that conformed to the 30th–70th height percentiles on the WHO Growth Charts for Canada (2014; www.whogrowthcharts.ca).

### Microsatellite genotyping

The GT microsatellite from the *GHR* V9 promoter region (chr5:42424274‐42424321 hg19 Genome Assembly) was genotyped using fluorogenic probes followed by capillary electrophoresis (ABI Genetic Analyzer 3730 XL; Life Technologies, Foster City, CA, USA) to discriminate allele size (ABI GeneMapper Version 4.1). Primers were designed to amplify a 155‐bp fragment containing a 24 GT repeat that was used as reference; deviation from this size allowed us to deduce the GT length of the different alleles (e.g. 157 bp = 25 repeats). The primers (Forward: 5′‐6‐FAM‐TCCTCCTTGCGAAGAAGTTG‐3′ and Reverse: 5′‐GTGTGATGGTTCGTCTGTCG‐3′) were used in a PCR with Phusion enzyme (ThermoFisher Scientific, MA, USA) and 3% DMSO at an annealing temperature of 60°C. Samples were then processed and analysed at the Genotyping Platform at Génome Québec (Montreal, QC, Canada).

We classified the alleles arbitrarily into three categories in order that the cut‐offs fall at ~±1S.D. around the median. Thus, the shortest (S) alleles were <24 repeats (representing ~16% of the individuals), the medium (M) alleles were 24–28 repeats (~68%), and the longest (L) alleles were >28 repeats (~16%) (Table S1)**.**

### SNP selection

Twenty‐seven SNPs with minor allele frequencies >5% were selected to span the *GHR* gene region from ~200 kb upstream of the major *GHR* (V2) transcriptional start site to ~120 kb downstream of the 3′UTR (Tables S3 and S4). We prioritized SNPs previously shown to be in association with height 22, 23, 39, transcription regulation 40 or disease risk (e.g. non‐small cell lung cancer 41 or prostate cancer 42).

### SNP genotyping and quality control

Genotyping was performed at Génome Québec using Sequenom iPLEX Gold Technology and the MassARRAY system (Agena Biosciences, San Diego, CA, USA). To assess robustness of the technology, ~20% of the total samples were replicated with 100% success rate. Quality controls were conducted prior to the analysis: all variants used for association analysis had a genotyping efficiency call rate >95% and showed no departure from Hardy–Weinberg equilibrium in combined controls and cases (*P* > 0.001) and in controls and cases separately. A table of the SNP probes is available on request.

### Statistical analysis

Allelic frequency calculations of the GT polymorphisms were performed using GraphPad Prism v7.0 software (La Jolla, CA, USA). Significance was calculated using 2 × 2 contingency tables and Fisher's exact tests to obtain *P*‐values, odds ratios (ORs) and 95% confidence intervals (95% CIs) as well as two‐tailed unpaired *t*‐tests and two‐way ANOVA. Bonferroni corrections were applied following Fisher exact tests to account for the multiple testing of the GT genotype categories (*P*‐corr < 0.01 [0.05/5]) and sex (*P*‐corr_sex_ < 0.005). ANOVA tests were followed by a Tukey *post hoc* test; *P* < 0.05 was considered significant. For case–control single marker as well as haplotype logistic regressions, we used the PLINK v1.07 software package. Results of the logistic regression were adjusted for sex but also calculated for each gender separately. The GT genotypes were also used as covariates in the regression analysis after coding them as dummy (binary) variables. Two genetic models were tested: additive and recessive 43. Measures of pairwise LD between SNPs (D' and *r* ^2^) and LD plots were computed using Haploview v4.2 (Broad Institute, Cambridge, MA, USA) and haplotype blocks were defined using the solid spine algorithm of LD (D'>0.8). Because of strong LD between certain SNPs in our panel, we used Haploview's Tagger software with a pairwise approach (*r* ^2^ > 0.8) to calculate that 18 SNPs represented the effective number of independent SNPs to use in the Bonferroni correction method for multiple comparison. For single marker analysis, the significant *P*‐value after correction by the number of effective SNPs was *P*‐corr < 0.0028 (0.05/18). For haplotype analysis, a permutation procedure was applied to correct for multiple comparisons.

## Results

### Specific GT microsatellite genotypes are associated with idiopathic short stature

**Figure 1:**
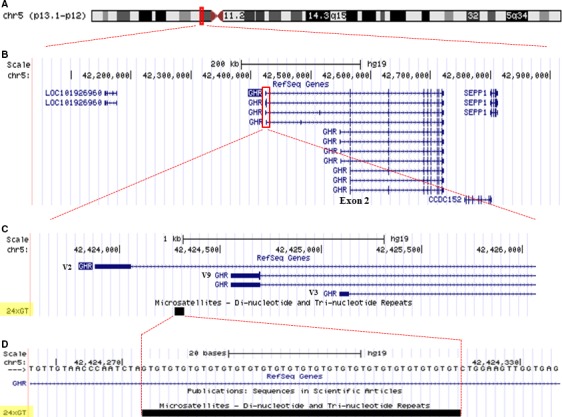
GT microsatellite localization in the *GHR* gene. (**A**) The *GHR* gene spans ~300 kb on the short arm of chromosome 5 close to the centromere. (**B**) Fourteen different *GHR* mRNAs encoding the full‐length GHR have been reported to date (not all first exons are shown here). The coding region is defined by exons 2–10 where exon 2 contains the translation start site. (**C**) Three of the exons transcribing ubiquitous *GHR* mRNAs (V2, V9, V3) are located within a 1.6 kb region of the 5′UTR. (**D**) A GT repeat lies within the V9 promoter and is polymorphic in the general population 36.

The *GHR* gene spans ~300 kb on chromosome 5 (Fig. 1A) 44, 45. The coding region is defined by exons 2–10 where exon 2 contains the translation start site 46. Fourteen different *GHR* mRNAs encoding the full‐length GHR have been reported to date 47, 48, 49, 50. They each have a unique 5′UTR, derived from different first exons, but all splice into the same site in exon 2, 11 bp upstream from the ATG translation start site and, thus, code for the same protein.

**Figure 2:**
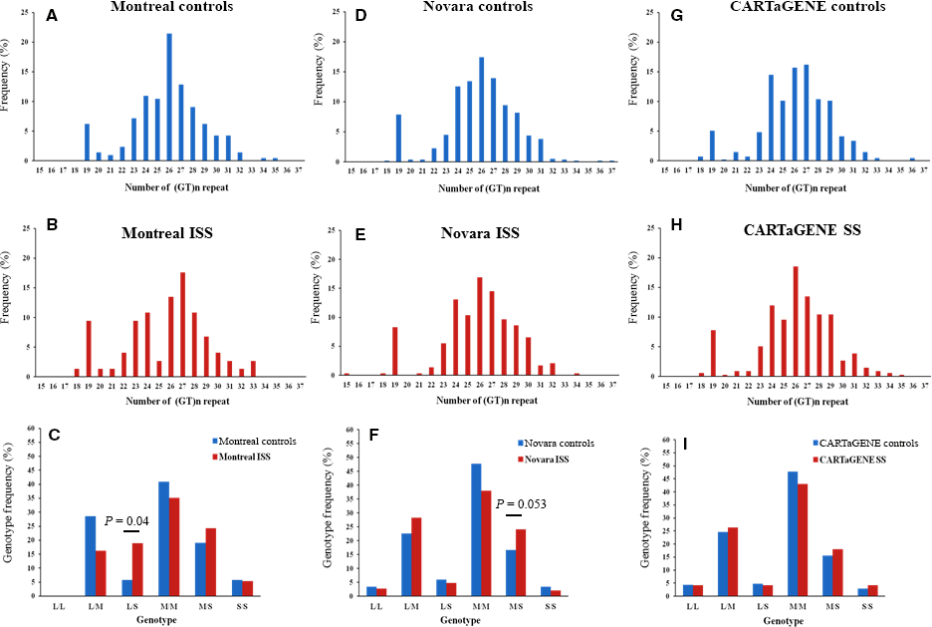
(GT)n satellite polymorphism allele distribution and genotype frequency in three different cohorts. (**A–C**) Montreal idiopathic short stature (ISS), (**D–F**) Novara ISS and (**G–I**) CARTaGENE SS *versus* normal height controls for each cohort. All cohorts displayed similar allelic distribution profiles with a median of 26 GT repeats. The Montreal ISS children showed a 3.3‐fold increase in L/S genotypes compared to their adult controls (Fisher exact test: *P* = 0.04). The Novara ISS cohort had 1.5‐fold more M/S genotype carriers than their controls (Fisher exact test: *P* = 0.053).

A (GT)_n_ repeat polymorphism is located in the proximal promoter of V9, one of the major ubiquitously expressing *GHR* 5′UTR exons (Fig. 1C and D**)**. This was genotyped in all three of our cohorts (Fig. 2). The allelic distribution profile was similar across the cohorts and showed a (GT) repeat number ranging from 15 to 37, with a median average at 26 and an isolated peak at 19 (Fig. 2A‐B, D‐E and G‐H). The repeat length cut‐offs for allelic categorization were defined arbitrarily as <24 repeats for the short (S) alleles, 24–28 for the medium (M) and >28 for the long (L) alleles; there were no significant differences in the frequencies of each category (S, M, L) among the three populations (Table S1). Following allele classification, we could attribute to each individual one of the six bi‐allelic genotypes: L/L, L/M, L/S, M/M, M/S or S/S. Genotype distribution frequencies are shown for each cohort in Figure 2C, F and I and Table S2.

The Montreal ISS children showed a nominally significant 3.3‐fold increase in L/S genotypes compared to their adult controls (Fig. 2C) (Fisher exact test: *P* = 0.04, OR = 3.85, 95% CI = 1.28–12.92). Interestingly, the Montreal ISS L/S genotype carriers had a significantly lower average height *z*‐score at diagnosis when compared to the non‐L/S children: −3.16 ± 0.79 *versus* −2.5 ± 0.37 (two‐tailed unpaired *t*‐test: *P* = 0.0026) (Fig. S1a). The ISS individuals also showed an association between the GT genotypes and the height *z*‐score at diagnosis, with the L/S genotype carriers being the shortest and the S/S the tallest, with a significant difference between the L/S and M/S carriers (one‐way anova and Tukey *post hoc* test: *P* = 0.026) (Fig. S1a). At the time of recruitment, the L/S genotype children tended to remain clustered at the lowest part of the growth curves, whereas the other genotype categories showed a percentage of individuals showing catch‐up growth (≥3rd percentile) (Fig. S1b). In the Novara replication ISS cohort, there were 1.5‐fold more M/S genotype carriers than in the normal height control children (Fig. 2F) (Fisher exact test: *P* = 0.053, OR = 1.64, 95% CI = 1.02–2.69). This difference was shown to be driven by the males (Fisher exact test: *P* = 0.017, OR = 2.26, 95% CI = 1.19–4.26) although this was only nominally significant after correction for multiple comparisons and sex (Fig. S2). There were no differences in average height scores at the time of diagnosis between participants of the M/S genotype compared to non‐M/S carriers; there was no information available on heights at the time of recruitment.

To validate the significance of our results in the two ISS cohorts, and because of the potential bias in the Montreal cohort due to a high proportion of female adult controls, we performed an analysis using a pool of control adults (composed of the CARTaGENE controls and a matching restricted set of Montreal adult controls [30th–70th percentile]). For the Montreal ISS group, the result confirmed the L/S genotype as being significantly associated with the ISS phenotype (Fisher exact test: *P* = 0.004, OR = 4.9, 95% CI = 1.88–13.39). For the Novara ISS cohort, there were still more males presenting with the M/S genotype than in the male controls (Fisher exact test: *P* = 0.038, OR = 2.1, 95% CI = 1.14–4.03) although this difference was only nominally significant.

We subsequently pooled the two ISS groups and used the same control adult pool. Again, there were more participants with the M/S genotype in the ISS group (Fisher exact test: *P* = 0.055, OR = 1.6, 95% CI = 1.02–2.52) and this was driven by the males (Fisher exact test: *P* = 0.0265, OR = 2.08, 95% CI = 1.14–3.96), similar to what we found in the original Novara ISS cohort. Interestingly, there were 2.3 times more females presenting with the L/S genotype in the pooled ISS cohorts compared to the pooled controls (9.8% *versus* 4.3%, respectively), but this result did not reach significance.

For the CARTaGENE SS cohort, no significant genotype frequency differences were observed between the short stature adult group and their controls even when the sexes were analysed separately. It is noteworthy that the homozygous L/L and S/S genotype frequencies were the lowest across all three cohorts and there were no significant differences between the sexes (Fig. 2C, F and I).

### Common variants in the *GHR* gene region are associated with idiopathic short stature

**Figure 3:**
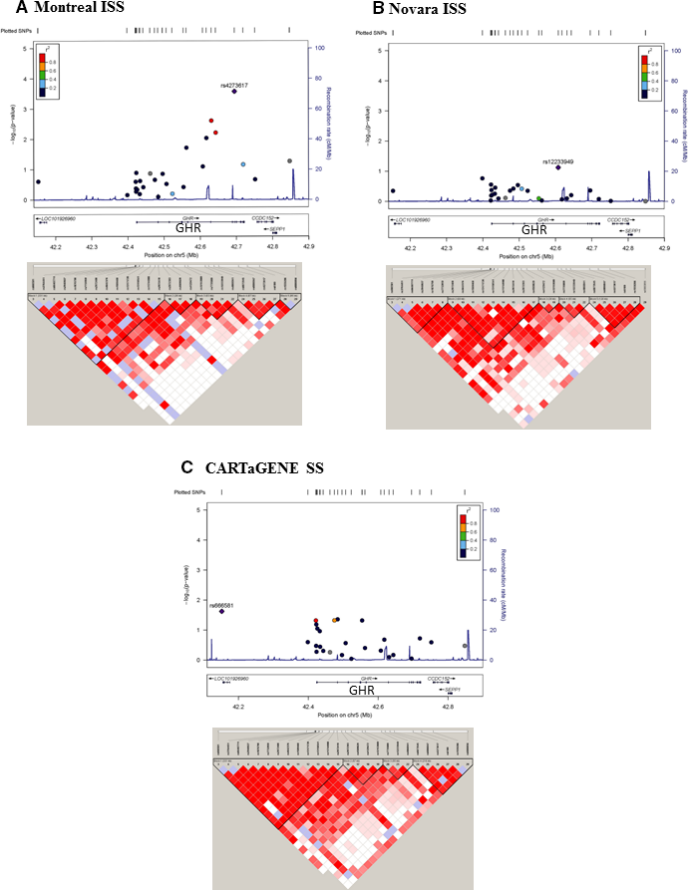
Association plot results (additive model) with short stature and haploblock structure of the *GHR* gene region in each cohort. Upper panels were generated using Locus Zoom 74, the 1000 Genomes LD European population (Nov 2014) and the hg19 genome build as references. Association results from logistic regression analysis (additive model) are shown as −log_10_ *P* values on the left *y*‐axis for the 27 SNPs genotyped. The most significant SNP is indicated as a purple diamond and the others are colour‐coded according to the strength of their LD relationship as measured by *r* ^2^. Local recombination rates are shown (blue line, scale on the right *y*‐axis). The intron–exon structures of local genes are provided below with direction of transcription and genomic coordinates in Mb. (**A**) In the Montreal idiopathic short stature (ISS) cohort, rs4273617 in intron 5 was the most significant (*P* = 3 × 10^−4^). (**B**) For the Novara cohort, rs12233949 in intron 2 was found to be the most significant (*P* = 0.075). (**C**) For the CARTaGENE cohort, rs666581 > 200 kb upstream of V2 was nominally significant (*P* = 0.0237). Lower panels: LD plots showing haplotype blocks for each of the populations. The heat map, based on D' values, was drawn using the SNP panel genotyped data with the Haploview software v4.2 using the solid spine algorithm.

To assess the association of common variants (MAF > 5%) in the *GHR* gene region with short stature, we selected a panel of 27 SNPs that were previously used in different association studies (Tables S3 and S4) 22, 23, 39, 40, 41, 42. Logistic regression analyses were conducted using additive or recessive penetrance models within each of our three cohorts and the most significant association results are shown in Tables 2 and 3 as well as Figure 3. Because height is sexually dimorphic in humans, we also analysed male and female groups separately.

**Table 2:** SNP association analysis of short stature in three different cohorts

SNP	Montreal ISS						Novara ISS						CARTaGENE SS					
	Risk allele†	Model‡	Sex§	*P* value¶	OR	95% CI	Risk allele	Model	Sex	*P* value	OR	95% CI	Risk allele	Model	Sex	*P* value	OR	95% CI
rs666581	C	A	B	0.248	3.4	0.43–26.47	C	A	F	0.352	1.5	0.63–3.73	A	A	B	**0.024**	1.9	1.09–3.29
rs3764451	C	A	B	0.695	1.2	0.53–2.58	C	A	F	0.140	1.7	0.85–3.28	C	A	M	0.232	1.4	0.80–2.54
rs66487711	T	A	B	0.253	3.3	0.42–26.06	T	A	F	0.549	1.3	0.52–3.43	C	A	B	**0.048**	1.7	1.0–2.97
rs2940927	G	R	F	0.106	2.7	0.81–8.99	A	R	F	0.255	0.7	0.32–1.36	G	A	F	0.184	1.3	0.88–1.99
rs1876790	C	A	M	0.330	1.6	0.62–4.1	C	R	B	**0.035**	0.3	0.11–0.92	T	A	F	0.053	1.8	1.0–3.35
rs7732059	G	A	B	0.129	1.8	0.85–3.7	G	A	B	0.421	1.2	0.82–1.61	C	R	F	0.062	3.2	0.9–10.8
rs2972400	A	A	M	0.352	1.6	0.6–4.13	A	R	B	**0.036**	0.3	0.11–0.93	G	A	F	0.053	1.8	1.0–3.35
rs4642376	T	A	M	0.491	1.4	0.54–3.58	T	R	B	**0.035**	0.3	0.11–0.92	G	A	F	0.053	1.8	1.0–3.35
rs1509460	A	R	F	0.106	2.7	0.81–8.99	C	R	F	0.231	0.6	0.31–1.33	A	A	F	0.146	1.4	0.9–2.06
rs13171720	T	R	F	0.253	5.2	0.31–89.05	C	A	F	0.622	1.1	0.67–1.94	T	R	F	0.260	2.7	0.48–15.12
rs13156541	G	A	F	0.106	2.1	0.83–7.02	C	A	F	0.627	1.1	0.71–1.78	G	R	B	0.081	2.1	0.91–4.70
rs11744988	C	A	B	0.220	3.6	0.46–28.53	C	A	B	0.441	1.3	0.71–2.19	T	A	B	**0.047**	1.7	1.01–3.02
rs2972419	A	R	B	0.562	0.5	0.06–4.70	A	R	B	**0.024**	0.2	0.07–0.83	G	A	F	**0.031**	2.0	1.06–3.61
rs2972393	A	R	B	**0.043**	0.3	0.10–0.96	G	A	B	0.288	1.2	0.88–1.53	G	A	F	0.170	1.4	0.88–2.07
rs4509029	G	A	F	0.129	1.8	0.84–3.90	G	R	B	0.298	1.3	0.79–2.12	G	A	F	0.095	1.4	0.94–2.19
rs4129472	A	A	F	0.288	1.9	0.59–6.04	A	A	F	0.233	1.4	0.8–2.46	G	R	F	0.260	2.7	0.48–15.12
rs12153009	A	R	F	0.101	5.6	0.72–43.54	A	R	B	0.229	0.5	0.16–1.54	A	R	B	**0.025**	3.1	1.15–8.15
rs7735889	A	A	B	**0.019**	2.3	1.15–4.61	A	A	F	0.475	1.2	0.76–1.81	G	R	B	0.081	2.1	0.91–4.70
rs12233949	C	A	B	**0.080**	1.7	0.94–3.12	G	A	M	0.054	1.8	1.0–3.12	C	R	B	**0.043**	2.8	1.04–7.50
rs4866941	G	A	B	**0.009**	2.8	1.29–6.09	A	A	M	0.421	1.2	0.77–1.86	A	A	B	0.208	1.2	0.89–1.75
rs4292454	T	A	B	**0.01**	2.3	1.22–4.48	C	A	B	0.383	1.1	0.85–1.51	C	R	M	0.06	2.1	0.95–4.58
rs6873545	T	A	B	**0.0025** ^*^	3.3	1.52–6.98	C	R	M	0.424	0.6	0.20–1.98	C	R	B	0.418	1.4	0.65–2.83
rs6886047	A	A	B	**0.0061**	2.9	1.35–6.12	T	R	M	0.239	0.5	0.13–1.67	T	R	B	0.544	1.3	0.60–2.66
rs4273617	A	A	B	**0.0003** ^*^	4.8	2.07–11.14	G	A	M	0.304	1.3	0.82–1.92	G	R	B	0.324	1.5	0.69–3.08
rs6180	C	A	B	0.069	1.7	0.96–3.08	A	A	M	0.660	1.1	0.74–1.61	A	A	B	0.189	1.2	0.91–1.65
rs1559286	G	A	M	0.197	2.3	0.64–8.47	G	R	B	0.689	0.6	0.06–6.12	T	A	B	0.255	1.4	0.77–2.62
rs6880056	A	A	B	0.054	1.9	0.99–3.71	A	R	M	0.685	1.5	0.24–8.9	T	A	M	0.192	1.4	0.85–2.25

ISS, idiopathic short stature; OR: odds ratio; 95% CI: 95% confidence intervals. ^†^Risk allele: major or minor allele. ^‡^Genetic model: A additive, R recessive. ^§^Sex: B Both, M Males, F Females. ^¶^ *P* values were calculated using logistic regression analysis. Highlighted in bold are tests nominally significant (*P* < 0.05) and those with asterisks are tests that remained significant after Bonferroni correction (*P* < 0.0028).

**Table 3:** SNP association analysis of idiopathic short stature *versus* pooled adult controls

SNP	Montreal ISS†						Novara ISS†						Montreal + Novara ISS†					
	Risk allele‡	Model§	Sex¶	*P* value††	OR	95% CI	Risk allele	Model	Sex	*P* value	OR	95% CI	Risk allele	Model	Sex	*P* value	OR	95% CI
rs666581	C	A	B	0.128	4.8	0.64–35.75	A	A	M	0.180	1.8	0.77–4.04	A	A	F	0.299	1.6	0.67–3.61
rs3764451	C	A	B	0.227	1.5	0.77–2.99	C	A	B	0.352	1.2	0.80–1.87	C	A	B	0.216	1.3	0.86–1.92
rs66487711	T	A	B	0.121	4.9	0.66–36.91	C	A	M	0.215	1.7	0.73–3.98	T	A	B	0.555	1.2	0.67–2.11
rs2940927	G	A	B	0.2	1.4	0.84–2.30	A	R	F	0.373	0.7	0.35–1.48	A	R	F	0.373	0.7	0.38–1.44
rs1876790	C	R	B	0.365	0.4	0.05–3.02	C	R	M	0.136	0.3	0.07–01.45	C	R	B	0.100	0.4	0.15–1.18
rs7732059	G	A	B	0.064	1.9	0.96–3.69	G	A	F	0.221	1.4	0.83–2.27	G	A	F	0.097	1.5	0.93–2.40
rs2972400	A	A	F	0.417	1.5	0.58–3.71	A	R	M	0.193	0.4	0.07–1.69	A	A	F	0.167	1.4	0.87–2.31
rs4642376	T	R	B	0.409	0.4	0.05–3.30	T	R	B	0.183	0.5	0.15–01.43	T	R	B	0.137	0.5	0.16–1.28
rs1509460	A	A	B	0.137	1.5	0.89–2.43	C	R	M	0.321	1.4	0.72–2.70	C	R	F	0.373	0.7	0.38–1.44
rs13171720	T	R	F	0.291	3.5	0.34–36.23	T	R	M	0.125	0.2	0.02–1.58	T	R	M	0.132	0.3	0.06–1.44
rs13156541	C	A	B	0.09	1.7	0.92–3.18	C	A	F	0.323	1.3	0.79–2.05	C	A	F	0.161	1.4	0.88–2.17
rs11744988	C	A	B	0.119	5.0	0.66–37.13	C	A	F	0.228	1.7	0.71–4.11	C	A	F	0.159	1.8	0.79–4.18
rs2972419	A	R	B	0.361	0.4	0.05–3.0	A	R	M	0.074	0.2	0.02–01.2	A	R	B	0.056	0.3	0.11–1.03
rs2972393	A	R	M	0.068	0.1	0.02–1.15	A	R	M	0.501	1.3	0.65–2.40	G	A	B	0.486	1.1	0.84–1.43
rs4509029	G	A	B	0.118	1.5	0.90–2.47	G	R	M	0.466	1.3	0.65–2.58	G	R	M	0.292	1.4	0.75–2.66
rs4129472	A	A	F	0.363	1.7	0.55–5.21	A	A	F	0.233	1.3	0.91–1.96	A	A	F	0.119	1.5	0.90–2.57
rs12153009	A	R	B	0.001*	7.1	2.22–22.82	G	A	M	0.255	1.4	0.79–2.39	A	R	B	0.119	2.2	0.82–5.90
rs7735889	A	A	B	**0.023**	2.2	1.11–4.16	G	R	F	0.282	1.7	0.66–4.15	A	A	F	0.389	1.2	0.79–1.83
rs12233949	C	A	B	**0.029**	1.9	1.07–3.40	G	A	M	**0.013**	2.1	1.17–3.76	G	A	M	0.162	1.4	0.87–2.29
rs4866941	G	A	B	**0.009**	2.7	1.28–5.54	A	A	M	0.164	1.4	0.87–2.25	G	A	F	0.464	1.2	0.76–1.81
rs4292454	T	A	B	**0.015**	2.1	1.16–3.85	C	R	B	**0.007**	2.0	1.21–3.25	C	R	B	**0.027**	1.7	1.06–2.75
rs6873545	T	A	B	**0.014**	2.3	1.19–4.47	C	R	B	0.408	0.7	0.32–1.59	C	R	B	0.157	0.6	0.25–1.25
rs6886047	A	A	B	**0.022**	2.2	1.12–4.15	T	R	M	0.261	0.5	0.12–1.76	T	R	B	0.101	0.5	0.22–1.15
rs4273617	A	A	B	0.001*	3.2	1.57–6.66	G	R	B	0.381	0.7	0.30–1.59	G	R	B	0.150	0.5	0.23–1.25
rs6180	C	A	F	0.118	2.0	0.84–4.80	C	A	M	0.709	1.1	0.71–1.64	C	R	B	0.551	1.1	0.73–1.80
rs1559286	G	A	M	0.106	2.3	0.84–6.29	G	A	M	0.628	1.2	0.53–2.84	G	A	M	0.303	1.5	0.71–3.03
rs6880056	T	A	B	0.089	1.7	0.93–2.96	A	A	B	0.656	1.1	0.74–1.60	T	R	B	0.446	1.5	0.52–4.41

OR, odds ratio; 95% CI, 95% confidence intervals; ISS, idiopathic short stature. ^†^For this analysis of the ISS groups separately as well as combined, the controls were a pool of Montreal adult controls with restricted height (*n* = 57) and the CARTaGENE adult controls (*n* = 207) (30th–70th percentile). ^‡^Risk allele: major or minor allele. ^§^Genetic model: A additive, R recessive. ^¶^Sex: B Both, M Males, F Females. ^††^ *P* values were calculated using logistic regression analysis. Highlighted in bold are tests nominally significant (*P* < 0.05) and those with asterisks are tests that remained significant after Bonferroni correction (*P* < 0.0028).

In the Montreal ISS cohort, two SNPs showed the strongest association with the ISS phenotype: (i) the A allele of rs4273617 in intron 5 (*P = 0.0003, OR = 4.8) and (ii) the T allele of rs6873545 in intron 3 (*P = 0.0025, OR = 3.3); these remained significant after correction for multiple comparisons (Table 2 and Fig. 3A). The latter SNP, rs6873545, has been used to tag a common deletion that removes *GHR* exon 3 23. During evolution, a homologous recombination event resulted in *GHR* alleles in ~35% of humans that differ by the deletion of exon 3 (3‐) 51. In this study, heterozygous (3+/3‐) individuals in the Montreal ISS cohort represented ~32% of the total cohort and no children were homozygous for the exon 3 deletion; these results were confirmed by multiplex PCR assays (data not shown).

When the Montreal ISS cohort was analysed using the larger pool of control adults, rs4273617 remained significant (A: *P = 0.001, OR = 3.2) and rs12153009 became significant, with a recessive penetrance (A: *P = 0.001, OR = 7.1) (Table 3). These two SNPs delimit a cluster of nominally significant SNPs spanning ~141 kb, from ~12 kb upstream of the V5 5′UTR exon to intron 5 of the *GHR* gene.

When we compared regression analyses for the Montreal and Novara ISS cohorts that used the same pooled adult control group (Table 3), two SNPs were replicated. Not only was there an increased risk associated specifically with the Novara males carrying the G allele of rs12233949 (*P* = 0.013, OR = 2.1) but the Montreal ISS risk allele (C) was now nominally significant (*P* = 0.029, OR = 1.9). In addition, rs4292454, also in intron 2, was significant for both ISS cohorts: the major T allele in the Montreal group was the risk allele (*P* = 0.015, OR = 2.1), while the minor C allele was the risk allele in the Novara ISS (*P* = 0.007, OR = 2.0). When the Montreal and Novara ISS cohorts were pooled and compared to the pooled adult controls (Table 3), the risk C allele of rs4292454 remained significant in the recessive model (*P* = 0.027, OR = 1.7). Thus, rs4292454 showed the strongest association with the ISS phenotype.

In addition, in the Novara ISS cohort, four SNPs were nominally significant in the recessive model; the same SNPs showed similar association strengths in the CARTaGENE SS female cohort in the additive model (Table 2). However, the allelic effects were opposite: minor alleles were protective in the Novara ISS cohort, whereas major alleles were increasing the risk in the CARTaGENE SS female cohort. The first three SNPs are clustered in the V2‐V9‐V3 region (Fig. 1C), whereas rs2972419 is located in intron 1 (Table S3). This last SNP has been associated with pygmy short stature, with a higher proportion of the ancestral allele (G) in the pygmy population compared to non‐pygmies, and with the derived allele (A) associated with taller stature 52. In the Novara cohort, the minor allele A showed a nominal association with the ISS phenotype but it had a protective odds ratio (*P* = 0.024, OR = 0.2). We looked for an effect on increased height in the Novara ISS children who were carrying the A allele (~45%), but did not observe a significant association with a higher height *z*‐score; in addition, there was no significant difference between the *z*‐scores of the three children with the AA genotype (−2.0 ± 0.11 [M ± S.D.]) and those of the total ISS group (−2.3 ± 0.4). Thus, it is unlikely that this SNP is a major influence on the ISS phenotype.

For the CARTaGENE SS females, the major G allele of rs2972419 was associated with an increased risk of being short (*P* = 0.031, OR = 2.0) (Table 2). However, the G allele was not associated with a smaller height *z*‐score in the female group. The most significant SNP in the CARTaGENE SS cohort was rs666581 (A allele: *P* = 0.024, OR = 1.9) which is located in the distal promoter ~200 kb upstream of the *GHR* V2 transcription start site (Fig. 3C, Table S3), downstream of a lncRNA.

In order to assess a possible GT‐SNP combinatorial effect, we conducted an independent set of logistic regression analyses adjusted for each GT genotype (data not shown). Using the GT genotypes as covariates did not alter the association results indicating that none of the GT genotypes have a significant effect in combination with our tested SNPs.

### Haplotype variation in the *GHR* gene region and differences in the ISS and SS cohorts

**Figure 4:**
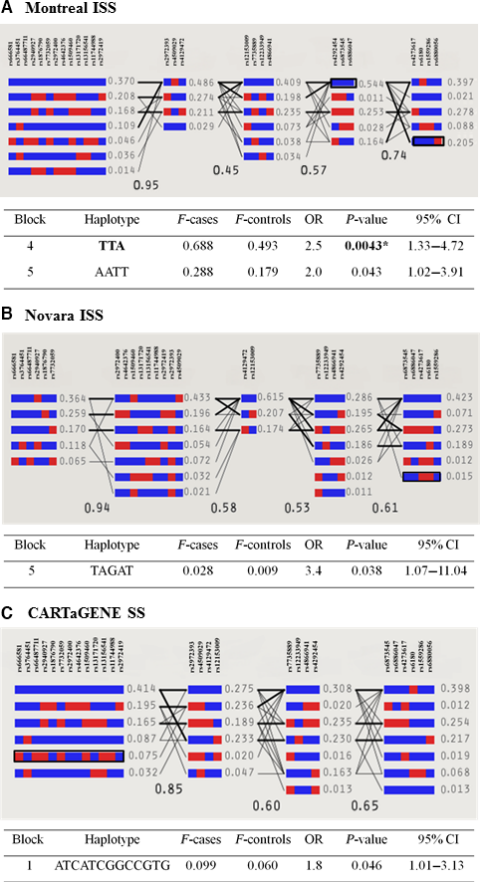
Haplotype analysis at the *GHR* locus. The upper panels represent the haplotype block structure in each of the populations studied. The haplotype blocks were determined using the solid spine algorithm from the Haploview software v4.2. Major and minor alleles are represented by blue and red squares, respectively. Frequency of each haplotype in cases and in controls is shown to the right of each haplotype and the D' value (indicating the level of linkage disequilibrium between two blocks) is also provided. Connections from one block to the next are shown for haplotypes of >10% frequency with thick lines and >1% frequency with thin lines. Each cohort displayed a specific haplotype block architecture in the *GHR* gene region, from five blocks in (**A**) Montreal idiopathic short stature (ISS) and (**B**) Novara ISS to four blocks in (**C**) CARTaGENE SS. The lower panels show *P* value, OR (odds ratio) and 95% CI results from haplotype logistic regression analyses. Frequencies (F) of each haplotype in cases and in controls are also provided. In the Montreal ISS cohort (**A**), association of a common risk haplotype, TTA in block 4 (OR = 2.5, *P* = 0.0043), remained significant after correction for multiple testing (*P < 0.05 after permutation test).

The *GHR* gene region has a relatively simple haplotype structure comprised of several large LD blocks 42. When we defined the LD structure of the *GHR* gene in the three cohorts based on our 27 genotyped SNPs, each group displayed a specific architecture, from five blocks in the Montreal and Novara ISS cohorts (Figs 3A‐B and 4A‐B) to four blocks in the CARTaGENE SS group (Figs 3C and 4C). The first two blocks span ~370 kb of the *GHR* gene locus, comprising the distal promoter and 5′ untranslated region. The third block (fourth for the Novara ISS cohort) contains the V5 promoter and V5 exon, exon 2 and intron 2. The last block (blocks 4 and 5 for Montreal ISS) starts from intron 3 and spans the remainder of the *GHR* coding region to downstream of the 3′UTR.

The two ISS cohorts showed significant haplotype variations in the last blocks: 4 and 5 for the Montreal ISS and 5 for the Novara ISS (Fig. 4A‐B). In the Montreal ISS cohort, the most significant risk haplotype was in block 4 (Fig. 4A): the TTA haplotype (formed by the major alleles of rs4292454, rs6873545 and rs6886047) represented half of the haplotypes in block 4 and was associated with an increased risk of 2.5 times for the ISS phenotype (*P* = 0.0043, OR = 2.5, 95% CI = 1.33–4.72; *P* = 0.04 after permutation test); it is noteworthy that the rs4292454 and rs6873545 major T alleles were associated with the ISS phenotype as single markers (Tables 2 and 3). A second risk haplotype in block 5, AATT, was significantly associated with the ISS phenotype (*P* = 0.043, OR = 2.0, 95% CI = 1.02–3.91) and included SNPs spanning from intron 5 (rs4273617) to ~120 kb downstream of the *GHR 3′UTR*. In the Novara ISS group, individuals presenting with the TAGAT haplotype in block 5 showed a nominally increased risk for being ISS (*P* = 0.038, OR = 3.4; 95% CI = 1.07–11.04); this haplotype was rare, as the frequency was 0.9% in the controls and 2.8% in the ISS group (Fig. 4B). In the CARTaGENE SS cohort, an at‐risk promoter haplotype in block 1 was nominally significant (*P* = 0.046, OR = 1.8, 95% CI = 1.01–3.13) with no difference between the sexes (Fig. 4C).

## Discussion

Our study examined the possible association of two types of genetic variations within the *GHR* gene locus, a GT microsatellite and SNPs, with the ISS phenotype. Our first goal was to investigate the highly polymorphic GT repeat located in the core promoter of the V9 5′UTR in a small exploratory cohort of ISS children and control adults recruited in Montreal. Genotyping demonstrated a high heterogeneity of the alleles, with lengths ranging from 15 to 37 repeats, confirming what we had previously shown in the general population 36. In addition, we identified a specific L/S genotype as being significantly more represented in the Montreal ISS cohort than adult controls. Interestingly, we also found that L/S children were the shortest group at the time of diagnosis and that none of the L/S children showed signs of catch‐up growth compared to non‐L/S children at the time of recruitment.

To validate these results, we examined the GT microsatellite in a larger cohort of ISS children from Novara, Italy 37, separately as well as in combination with our Montreal ISS group. In the Novara cohort, there were 1.9‐fold more M/S males within the ISS group than in the control male children; when we combined the two ISS groups and compared them with adult controls, this result was confirmed but was only nominally significant. In contrast, the L/S genotype was more frequent in the female ISS group, although this result did not reach significance. The absence of an association of a GT genotype with short stature in the CARTaGENE adult cohort suggests a possible specificity of this polymorphism for the ISS phenotype.

Although our study is the first to indicate potential sex‐specific differences for association of a microsatellite in *GHR* with the ISS phenotype, a CA repeat in the *IGF‐1* gene promoter has previously been associated with short stature as well as constitutionally tall stature in a sex‐specific manner 53, 54. In the future, it would be of interest to investigate, in parallel, the *GHR* GT and the *IGF‐I* CA repeat polymorphisms (and potentially others within the *GH‐IGF‐1* axis genes) to determine whether there are separate or combinatorial mechanisms regulating the growth of ISS children.

Microsatellites represent ~3% of the human genome, with CA/GT repeats the second most common form. Promoter microsatellites, by expanding and contracting in length, are often polymorphic, mainly due to slippage during DNA replication 55. They are found non‐randomly distributed at a high density within promoters and, the more proximal they are to the transcription start site, the more likely it is that they are conserved 56. Our V9 GT microsatellite is highly conserved in primates as well as the cow, the rat, the mouse and even the opossum, although the number of GT repeats varies across these species (UCSC browser).

Because their main location is within non‐coding DNA, microsatellites have been traditionally considered to be neutral markers. However, many recent reports have shown that these polymorphic repeats can modulate gene transcriptional activity, particularly of genes involved in the regulation of growth and development (e.g. *PAX‐6, COL1A2, gamma gene IV52S*) 30, 57, 58, 59. Modulation of promoter activity by these microsatellites can ultimately lead to phenotypic alteration and disease states (e.g. *NRAMP1, COL1A2, GRIN2A, STAT6, heme‐oxygenase 1*) 31, 32, 33, 34, 35. For example, specific GT genotypes in the promoter of the *HO‐1* (*heme‐oxygenase 1*) gene have been linked to altered transcriptional activity 60, 61, due to modulation of flanking response elements 35, and are associated with increased risk for several different diseases (cardiovascular disease, rheumatoid arthritis, type 2 diabetes mellitus, cancers) 62, 63, 64, 65. The cis‐regulatory effects may also be explained, in part, by the intrinsic property of the GT repeat sequences to form an alternative Z‐DNA structure, primarily due to the alternation of purine–pyrimidine nucleotides 66, 67. The left‐handed Z‐DNA motif has been found to modulate promoter activity, likely due to the binding of specific Z‐DNA binding proteins 31, 68. We are presently investigating the biological significance of the V9 GT microsatellite and its potential impact on *GHR* expression.

Many investigations have examined the SNPs associated with variation in height, primarily through GWAS studies of adults 17, 18, 19, 20, 21, 22. In our study, we focused on evaluating the possible contribution of common variants found within the *GHR* locus in ISS children and SS adults at the extreme tail of the height distribution. We selected a panel of 27 common SNPs (average MAF~27%) used in previous association studies that showed a strong potential for having a functional impact on *GHR* gene regulation 23, 39, 40, 41, 42. Indeed, we found two, rs6873545 and rs4273617, for which the major alleles were significantly associated with ISS in our Montreal cohort. Rs6873545 has been used as a tag for exon 3 deletion in *GHR*; the minor 3‐ allele codes for a GHR that is missing 22 amino acids in its extracellular domain, N‐terminal to the GH binding domain 51. Although there have been controversies regarding the physiological role of the exon 3‐ polymorphism, its major association appears to be restricted to an increased baseline height and growth velocity during the first year of GH treatment in GH‐deficient (GHD) children; there was no relationship in non‐GHD children, including ISS children 69. Our study is in line with these results: the minor exon 3‐ allele was not associated with the ISS phenotype in the Montreal or Novara children (even when they were combined) or the SS phenotype in the CARTaGENE adults.

Interestingly, rs12233949 showed the same directional effect across the three different cohorts, even though we saw the opposite risk allele in the Novara ISS cohort. Baas *et al*. 70 have reported similar opposite associations for three *SLC2A1* SNPs in two highly comparable populations. In addition, Lin *et al*. 71 have observed what they called a ‘flip‐flop’ phenomenon for SNPs in the *COMT* and *GAPDH* genes in two different ethnic populations. This type of ‘flip‐flop’ association is difficult to explain but may be due to different genetic backgrounds and haplotypes within each ethnic group 71.

In addition, rs4292454 was consistently associated with ISS in the two ISS cohorts, alone or combined, when compared to the pooled adult controls. Both rs4292454 and rs12233949 are located in intron 2 and are in low LD (*r* ^2^~0.35 in the CEU population), suggesting that this region could be a site of variation associated with ISS. *GHR* intron 2 spans ~63 kb and contains multiple DNase I hypersensitive sites and ChIP‐validated binding sites for transcription factors, including for CTCF, a polyfunctional regulator that can mediate long‐range chromatin looping and has been implicated in transcriptional regulation 72.

In order to investigate the possibility of a multiple marker effect associated with ISS and SS, we undertook a haplotype analysis in our three cohorts. Our results showed a consistent association of the risk haplotypes located in the 3′ haploblocks of the *GHR* gene with the ISS phenotype. These haplotypes encompass intron 2 to ~120 kb downstream of the *GHR* 3′UTR. In a previous haplotype‐based analysis, the same region was shown to be associated with prostate cancer risk in elderly men and with decreased BMI in the controls 42. Interestingly, in the CARTaGENE cohort, the risk haplotype for the adult SS phenotype showed a shift to the 5′ region of the *GHR* locus, >200 kb upstream of V2. The results of the haplotype analyses suggest distinct genetic variations for the ISS *versus* SS phenotype and potentially different mechanisms for regulating *GHR* expression.

## Conclusion and limitations

The goal of this study was to assess the potential role of the *GHR* gene in ISS by examining two different types of genetic polymorphisms. Our case–control analysis revealed a significant association of a GT microsatellite in the *GHR* promoter with ISS, while SNP analysis showed a consistent direction of effect for one common variant in intron 2 of *GHR* (rs4292454). There are two major limitations of this study: the size of the ISS cohorts and the fact that the majority of our ISS participants were recruited before they had achieved their final height. It is also important to recognize that, because the mean height of some of our ISS individuals at diagnosis was ˂1^st^ percentile, their short stature phenotype may not be explained by only common variants in the *GHR* gene locus. When Chan *et al*. 73 examined cohorts of extreme short and tall stature, they found that common genetic variants associated with height in the general population are also associated with height of individuals at the ~1^st^ percentile but are less predictive at the most extreme end (~0.25 percentile, <−2.8 SDS). Finally, even though the participants of our cohorts were unrelated and the majority were from European ancestry, we cannot exclude the possibility of a certain level of stratification that was not taken into account in this analysis. These findings underline the necessity to look in larger ISS cohorts for additional genetic contributors (e.g. rare variants, additional tandem repeats, structural polymorphisms, gene–gene or gene–environment interactions) to better define the association of the *GHR* gene with the idiopathic short stature phenotype.

## Conflict of interest

The authors do not have anything to disclose.

## Supporting information


**Fig. S1** GT genotype association with height *z*‐score in the Montreal ISS cohort.

**Fig. S2** (GT)_n_ microsatellite polymorphism allele genotype frequency in the Novara cohort by gender.

**Table S1** Relative frequencies (%) for each GT allele category for each cohort.

**Table S2** Number of individuals per cohort and GT genotypes.

**Table S3** SNP panel information.

**Table S4** Minor allele frequencies (MAF) for each population (controls + cases).
