# Supplementary figures and images for: Genetic variations at the human growth hormone receptor (GHR) gene locus are associated with idiopathic short stature
Source: J Cell Mol Med. 2017 May 29;21(11):2985–99. doi: 10.1111/jcmm.13210 (PMC5661101; doi:10.1111/jcmm.13210)

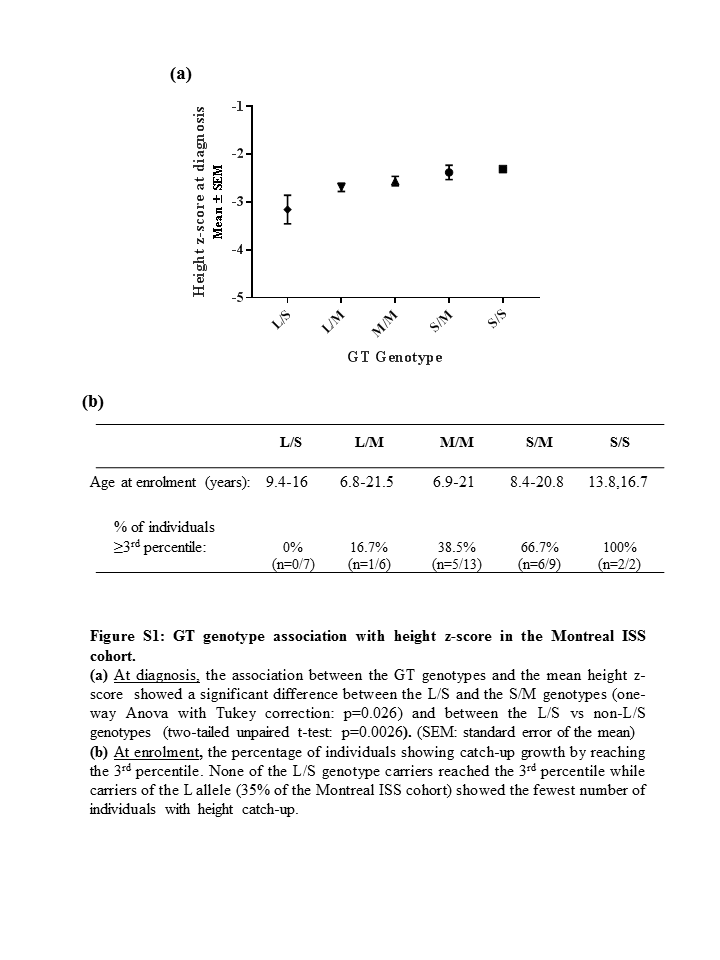

Supplement: Supplementary file 1 — Fig. S1 GT genotype association with height z‐score in the Montreal ISS cohort. [file JCMM-21-2985-s001.TIF]

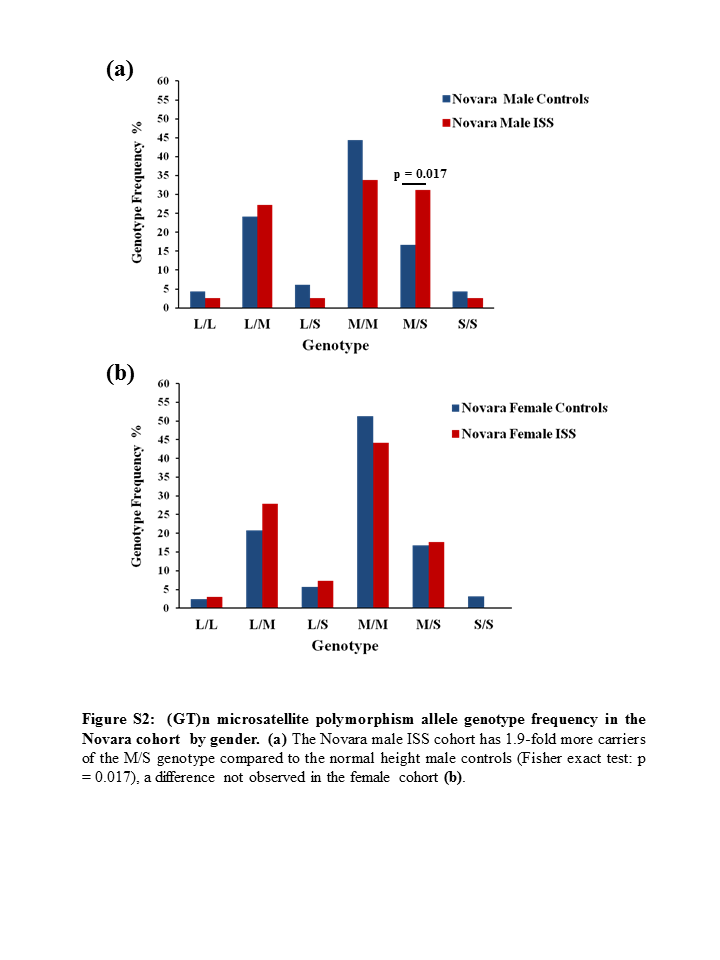

Supplement: Supplementary file 2 — Fig. S2 (GT)n microsatellite polymorphism allele genotype frequency in the Novara cohort by gender. [file JCMM-21-2985-s002.TIF]

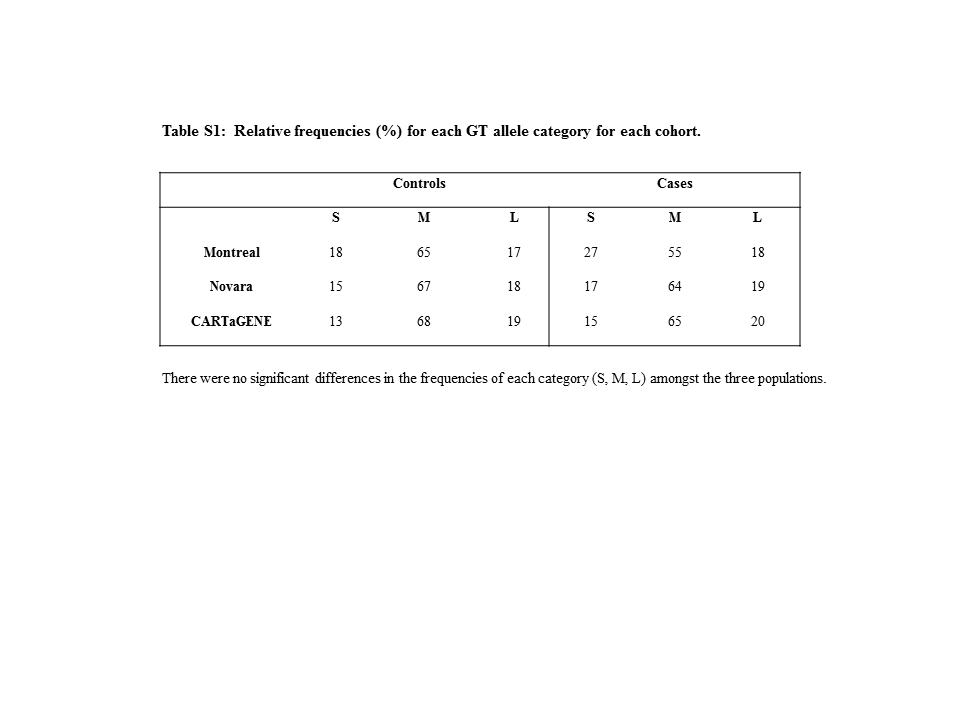

Supplement: Supplementary file 3 — Table S1 Relative frequencies (%) for each GT allele category for each cohort. [file JCMM-21-2985-s003.TIF]

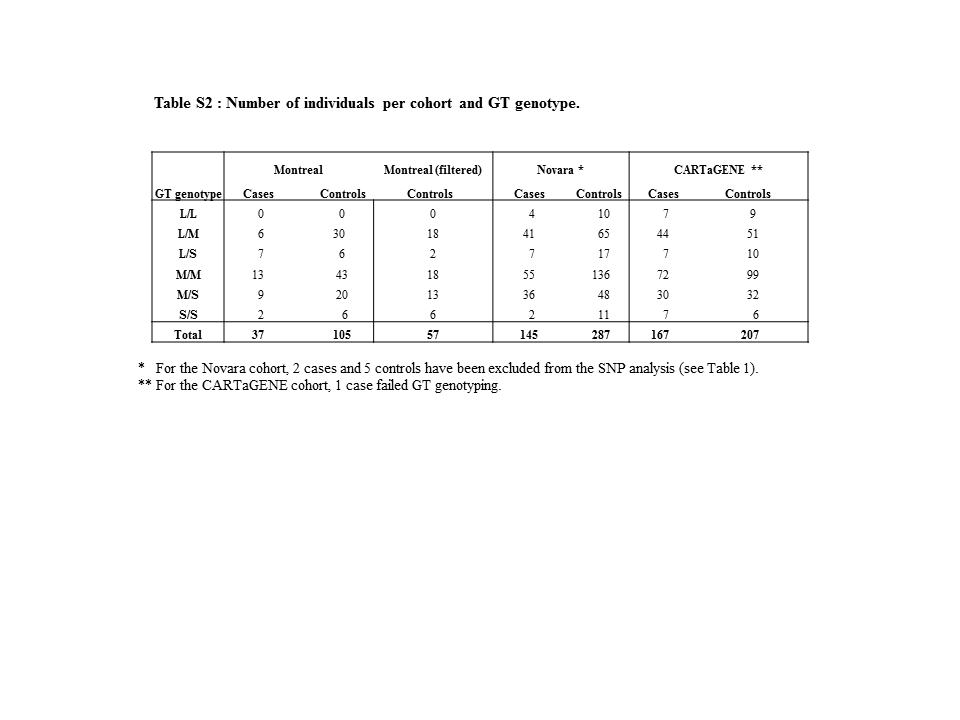

Supplement: Supplementary file 4 — Table S2 Number of individuals per cohort and GT genotypes. [file JCMM-21-2985-s004.TIF]

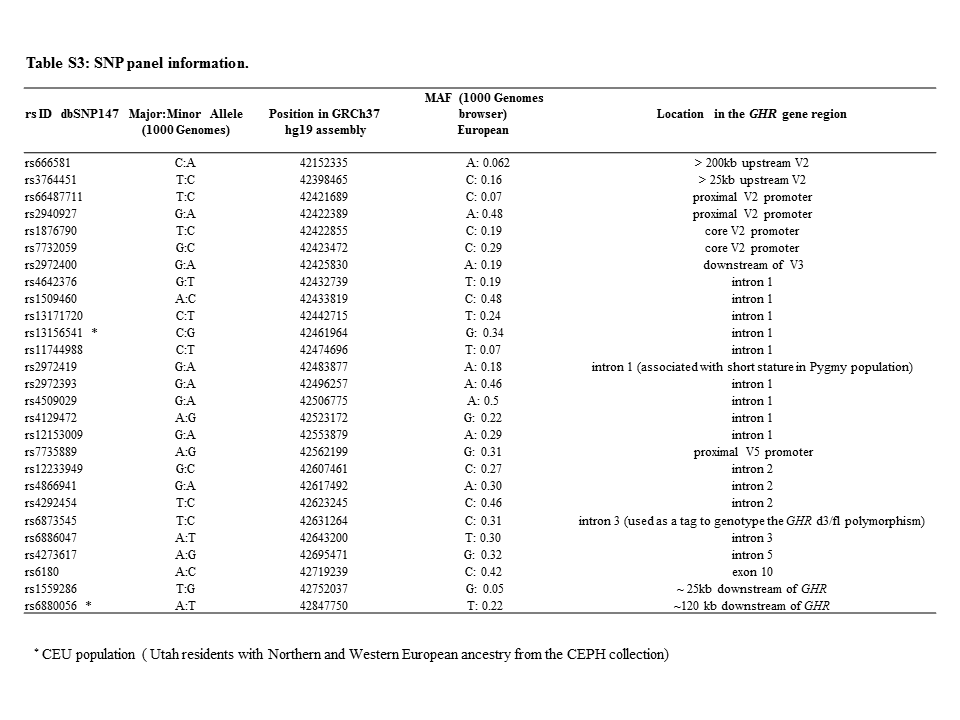

Supplement: Supplementary file 5 — Table S3 SNP panel information. [file JCMM-21-2985-s005.TIF]

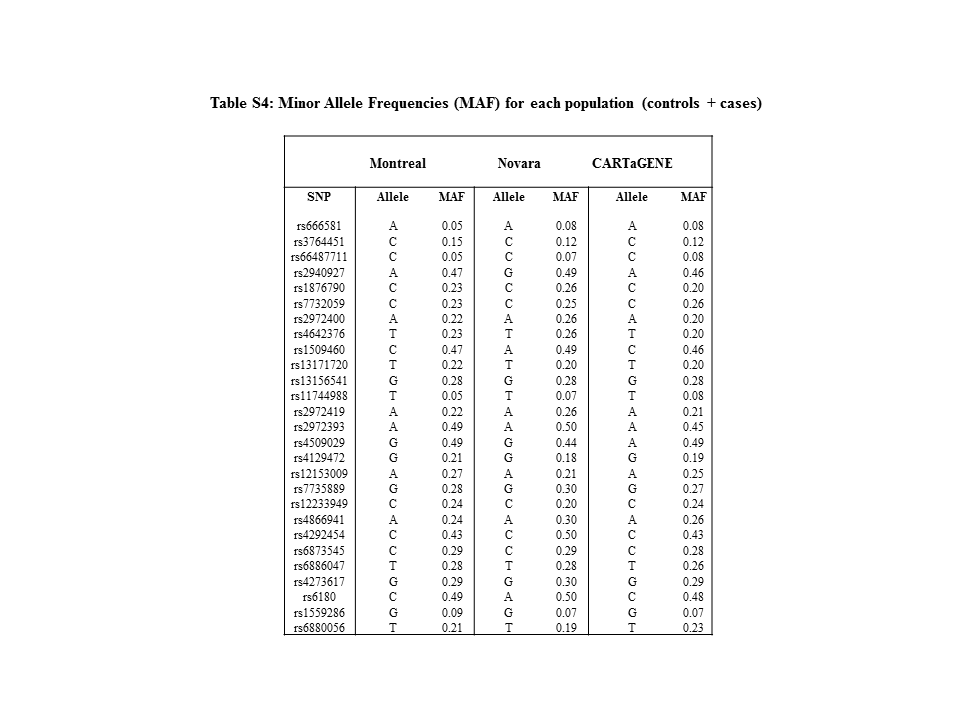

Supplement: Supplementary file 6 — Table S4 Minor allele frequencies (MAF) for each population (controls + cases). [file JCMM-21-2985-s006.TIF]

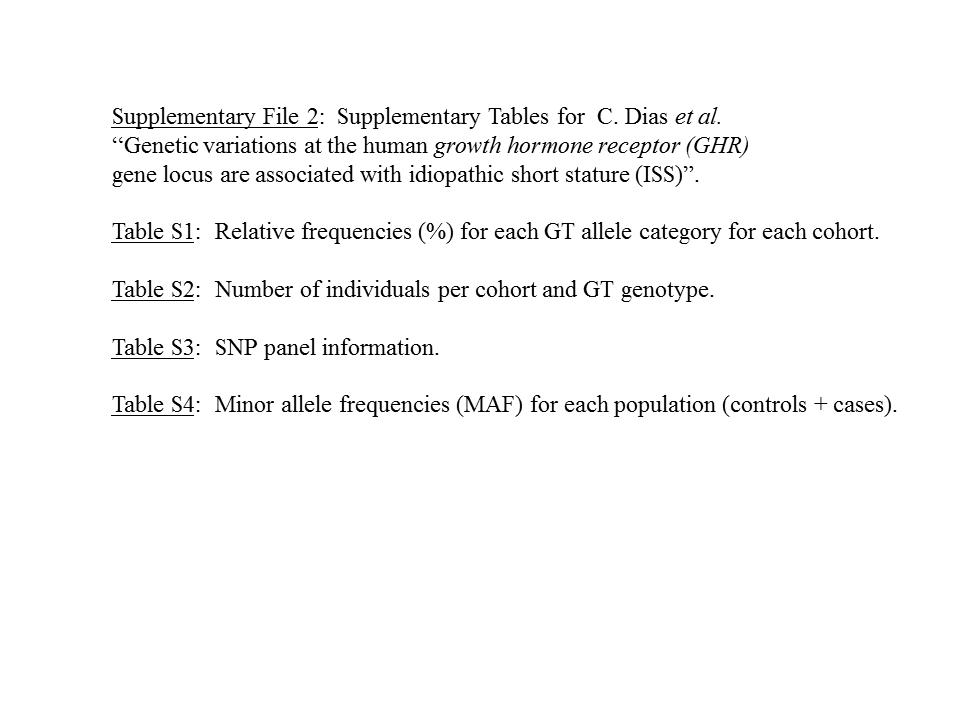

Supplement: Supplementary file 7 [file JCMM-21-2985-s007.TIF]
